# Fibrinogen Deposition on Silicone Oil-Infused Silver-Releasing Urinary Catheters Compromises Antibiofilm and Anti-Encrustation Properties

**DOI:** 10.1021/acs.langmuir.2c03020

**Published:** 2023-01-20

**Authors:** Shuai Zhang, Xiao Teng, Xinjin Liang, Geoffrey Michael Gadd, Colin Peter McCoy, Yuhang Dong, Yimeng Wang, Qi Zhao

**Affiliations:** † School of Pharmacy, Queen’s University Belfast, BT9 7BL, Belfast, United Kingdom; ‡ School of Life Sciences, University of Dundee, DD1 5EH, Dundee, United Kingdom; § School of Mechanical and Aerospace Engineering, Queen’s University Belfast, BT9 AG, Belfast, United Kingdom; ∥ State Key Laboratory of Heavy Oil Processing, Beijing Key Laboratory of Oil and Gas Pollution Control, China University of Petroleum, Beijing102249, China; ⊥ School of Science and Engineering, University of Dundee, DD1 4HN, Dundee, United Kingdom

## Abstract

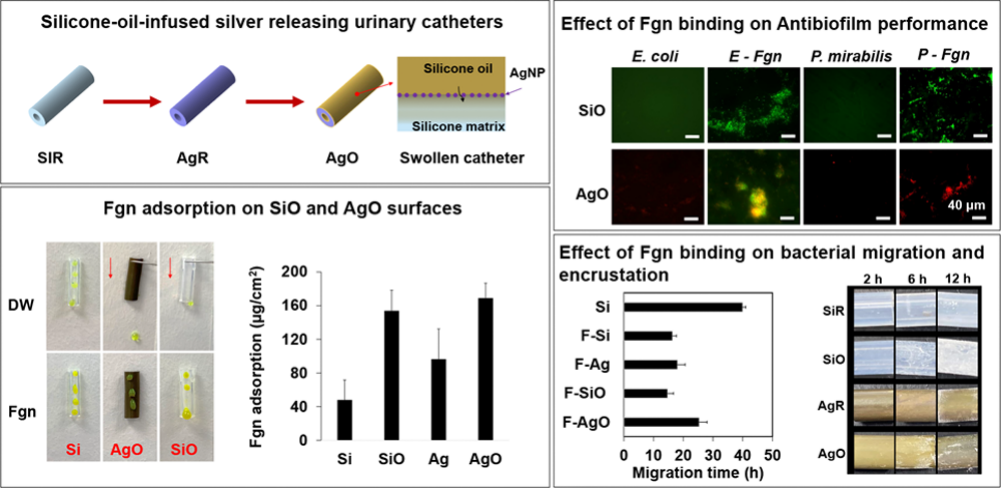

Slippery silicone-oil-infused (SOI) surfaces have recently emerged as a promising alternative to conventional anti-infection coatings for urinary catheters to combat biofilm and encrustation formation. Benefiting from the ultralow low hysteresis and slippery behavior, the liquid-like SOI coatings have been found to effectively reduce bacterial adhesion under both static and flow conditions. However, in real clinical settings, the use of catheters may also trigger local inflammation, leading to release of host-secreted proteins, such as fibrinogen (Fgn) that deposits on the catheter surfaces, creating a niche that can be exploited by uropathogens to cause infections. In this work, we report on the fabrication of a silicone oil-infused silver-releasing catheter which exhibited superior durability and robust antibacterial activity in aqueous conditions, reducing biofilm formation of two key uropathogens *Escherichia coli* and *Proteus mirabilis* by ∼99%, when compared with commercial all-silicone catheters after 7 days while remaining noncytotoxic toward L929 mouse fibroblasts. After exposure to Fgn, the oil-infused surfaces induced conformational changes in the protein which accelerated adsorption onto the surfaces. The deposited Fgn blocked the interaction of silver with the bacteria and served as a scaffold, which promoted bacterial colonization, resulting in a compromised antibiofilm activity. Fgn binding also facilitated the migration of *Proteus mirabilis* over the catheter surfaces and accelerated the deposition and spread of crystalline biofilm. Our findings suggest that the use of silicone oil-infused silver-releasing urinary catheters may not be a feasible strategy to combat infections and associated complications arising from severe inflammation.

## Introduction

Catheter-associated urinary catheter infections (CAUTIs) and encrustation remain two major problems in the care of patients undergoing long-term indwelling bladder catheterization. Upon being inserted into the urethra, the catheter acts as a bridge for the entry of bacteria into the bladder and simultaneously impairs normal defense mechanisms, which can result in bacteriuria and associated bloodstream infections as well as systemic dissemination with a 30% mortality rate.^1,2^ Standard treatments including catheter removal and antibiotic therapy are ineffective as the biofilms formed on the catheter surface and bladder wall protect uropathogens from antibiotics and the host immune response and can persist as a continuing focus of infection.^3^ Over recent decades, attempts have been made to endow catheters with antibacterial or antiadhesion functions by coating/impregnating catheter surfaces with, e.g., antibiotics, silver alloy, polytetraflouroethane (PTFE), and hydrogels.^4^ These commercially available catheters have achieved varied success in vitro but recent clinical research suggested that they were unable to significantly reduce symptomatic CAUTIs, when compared with standard catheters.^5^ The rapid release of antibiotics (e.g., nitrofural) and silver from catheters can only temporarily delay the onset of infection while intermittent and weak urine flow over PTFE or hydrogel-coated catheter surfaces was not sufficient to wash off the adhered bacteria.^6^ To solve this problem, we recently reported the use of a silver-doped superhydrophobic (SH) coating to combat CAUTIs by constructing hierarchical micro/nano surface structures with low surface energy to minimize surface-bacteria contact.^7^ The SH coating was able to effectively retard biofilm formation under dynamic conditions, but the increased surface roughness may compromise patient comfort.

Unlike traditional solid-state surfaces, a surface with dynamic features may avoid the permanent interactions between solid surfaces and bacteria, regardless of the time scale, thereby disrupting bacterial adhesion and inhibiting biofilm formation.^8^ Inspired by the Nepenthes pitcher plant, a slippery liquid-infused porous surface (SLIPS) was first introduced by Aizenberg’s group based on infusing textured or porous surfaces with a liquid lubricant.^9^ Recently, silicone-oil-infused silicone materials have emerged as an effective, nontoxic, and long-lasting candidate for resisting bacterial attachment and show great promise as anti-infection surfaces for urinary catheters.^10−12^ Moreover, because of the ultralow friction coefficient, the infusion of silicone oil into all-silicone catheters could improve catheter lubrication, allowing for improved patient comfort during insertion. However, previous studies have revealed that the existing silicone-oil-based SLIPs do not completely inhibit bacterial colonization as certain types of bacteria can breach the liquid barriers and establish “beachheads” on underlying surfaces, enabling bacterial colonization, proliferation, and biofilm formation.^13,14^

To address this limitation, we reasoned that the antibiofilm properties of silicone oil-infused catheters could be improved by combining with active antibacterial agents, leading to a synergistic function via passively disrupting bacterial colonization while actively killing the planktonic and attached bacteria. In this study, in order not to compromise the “slippery” character, the silicone catheters were predeposited with silver nanoparticles before oil infusion. Moreover, most recent findings in human urinary catheterization have revealed that long-term catheterization may induce severe bladder inflammation, resulting in the release of host fibrinogen (Fgn), which can deposit on catheter surfaces and work as a scaffold to bind uropathogens, promoting bacterial attachment and biofilm formation.^15^ However, few studies have reported the antiadsorption performance of silicone-oil-infused coatings against Fgn and the effect of Fgn deposition on their antibiofilm and antiencrustation properties. Herein, we report the fabrication of silicone-oil-infused silver-releasing urinary catheters via a two-step method. The stability and durability of the coating were examined under dynamic aqueous conditions, and the swelling ratios, contact angle hysteresis (CAH), and silver leaching were investigated for up to 7 days. Fgn adsorption on different surfaces was compared and analyzed, and the effect of Fgn adsorption on antibacterial performance was assessed using *Escherichia coli* ATCC 25922 and *Proteus mirabilis* ATCC 51286. The effect of Fgn binding on bacterial migration and encrustation was also investigated using a bridge model and an in vitro encrustation model, respectively.

## Materials and Methods

### Materials

Medical grade silicone sheets (thickness: 1 mm) were purchased from Goodfellow, Ltd. (Cambridge, UK), and cut into disks with a diameter of 1 cm. All-silicone urinary catheters were purchased from Bard Ltd. (West Sussex, U.K.) and cut into segments 1 cm long. LIVE/DEAD BacLight Bacterial Viability Kit L13152, Alexa Fluor 488 Phalloidin, and 4′,6-diamidino-2-phenylindole, dihydrochloride (DAPI) were purchased from Thermo Fisher Scientific (Paisley, U.K.). *Escherichia coli* ATCC 25922 *(E. coli)* and *Proteus mirabilis* ATCC 51286 *(P. mirabilis)* were obtained from the American Type Culture Collection (ATCC, Buckinghamshire, U.K.). Low swarm agar (LSW) and Mueller Hinton Agar (MHA) were purchased from Oxoid Ltd. (Hampshire, U.K.). Other chemicals used in this study were purchased from Merck Life Science UK, Ltd. (Dorset, U.K.) and used without further purification.

### Coating Preparation

**Figure 1:**
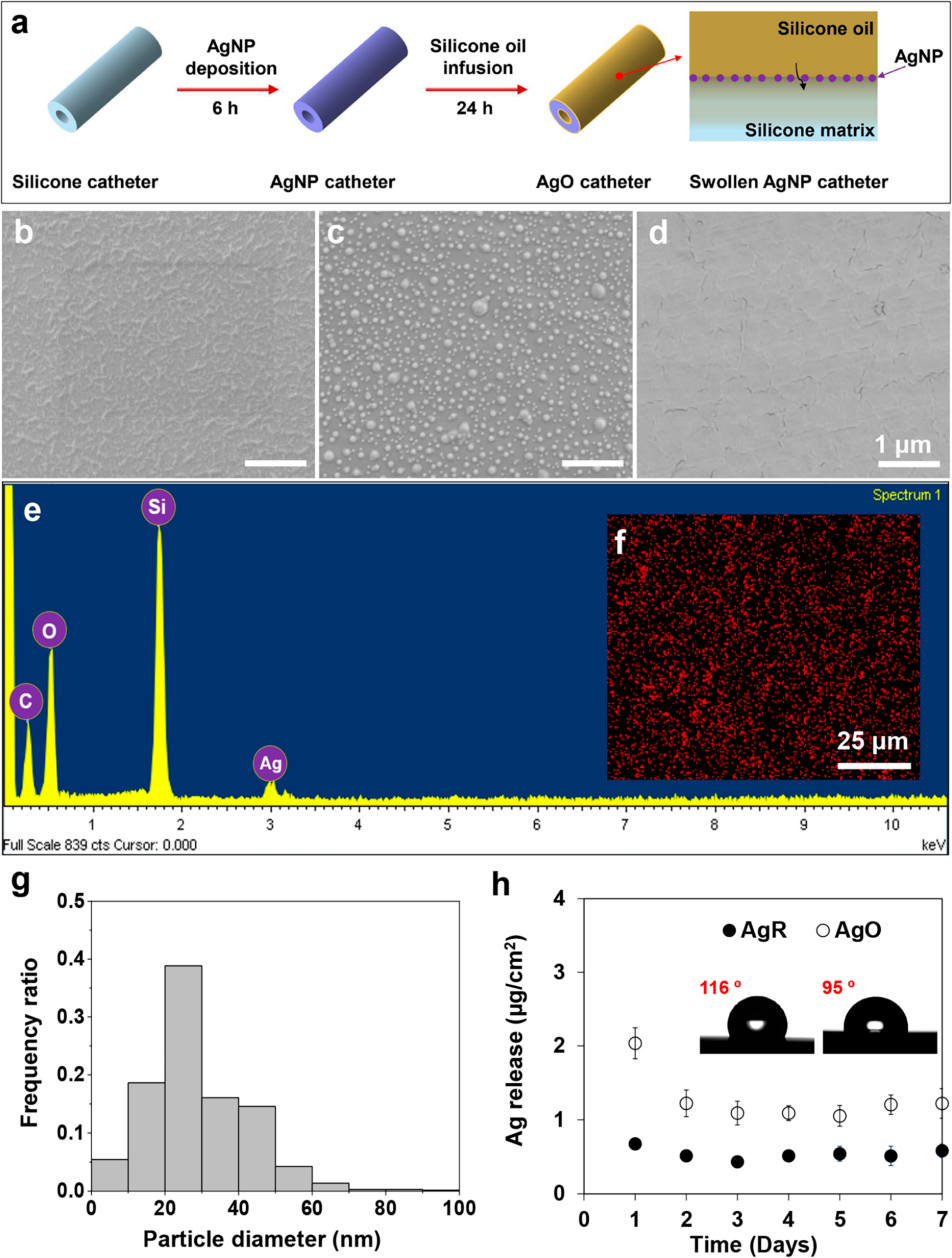
Fabricating process of AgNP-doped, liquid-infused AgO catheters; SEM images of (b) SiR, (c) AgR, and (d) AgO surfaces (scale bars correspond to 1 μm); EDX spectrum of the (e) AgO surfaces; (f) EDX mapping of silver of the AgO surface; (g) size distribution of the AgNPs in the SEM image in panel (c); (h) silver release profiles from AgR and AgO over time (*n* = 3, bars represent standard deviation of the mean).

To ensure good coating strength, all the silicone disks and catheter segments were sequentially rinsed with ultrasonication in ethanol, acetone, and deionized water (DW). The coating fabrication process is illustrated in Figure 1a (presented later in this work). For AgNP deposition, the samples were immersed into a colloidal silver suspension containing 5 mL/L Tween 20, 1.0 g/L sodium saccharine, 0.5 g/L silver nitrate, and 10 mL/L *N*,*N*,*N*′,*N*′ - tetramethylethylenediamine and maintained at 70 °C.^16^ After 6 h, the samples were taken out and ultrasonically cleaned in ethanol, acetone, and DW, aired dried, and stored in darkness at room temperature before further treatment. To prepare silicone-oil-infused samples, the AgNP-coated (AgR) and uncoated disks and catheters (SiR) were immersed in 10 cSt silicone oil for 24 h at 80 °C. After swelling, the samples were taken out and the excess oil was removed by gently wiping with a medical tissue. The silicone-oil-infused SiR (SiO) and AgR (AgO) were stored at room temperature for 24 h before further use.

### Characterization

The surface morphologies and chemical compositions of the samples were characterized using field-emission scanning electron microscopy (FE-SEM) (Model JSM-6500F, JEOL, Tokyo, Japan) and energy-dispersive X-ray analysis (EDX) systems (Model X-Stream-2/micsF+, Oxford Instruments, Oxford, U.K.), respectively. The size distribution of the AgNPs was analyzed using ImageJ (LOCI, University of Wisconsin, Madison, WI, USA). The absorption spectra of the proteins were obtained using attentuated total reflection–Fourier transform infrared (ATR-FTIR) spectroscopy (Single Reflection ATR, PIKE MIRacle, Madison, WI, USA). The thickness of the AgNP coating was determined by ellipsometry, using a Gaertner Scientific Stokes ellipsometer at a 70° incident angle (*n* = 6). The light source is a He–Ne laser (λ = 632.8 nm). Thickness was calculated using the manufacturer’s GEMP software. The surface roughness was determined by atomic force microscopy (AFM) (Asylum Research Cypher S, Oxford Instrument, Oxford, U.K.). All AFM measurements were operated in a tapping mode using a diamond-like carbon (DLC)-coated silicon cantilever (spring constant, 320 kHz). The arithmetic mean roughness (*R*_a_) values were obtained from images of scan size 3 μm × 3 μm captured at three different positions. The thickness of the oil layer was calculated according to Chen.^10^ Contact angles (CAs) were measured using a sessile drop method (drop volume: 4 μL) at room temperature with a tensiometer (Theta Flow, Bolin Scientific, Sweden). The advancing and receding CAs were measured while the probe fluid was added to and withdrawn from the drop, respectively. The drop/dispense rate was controlled at 0.4 μL/s. The release of Ag^+^ from AgR and AgO was monitored by inductively coupled plasma–optical emission spectrometry (ICP-OES) (Model Agilent 5100, Agilent, Santa Clara, CA, USA). The swelling ratio (*R*) of the sample after oil infusion was calculated as *R* = *M*_i_/*M*_o_ (where *M*_i_ is the mass after oil infusion and *M*_o_ is the mass before oil infusion).

### Stability Tests

To investigate the durability of the coatings in an aqueous system, the silicone oil-infused samples (*n* = 3) were vertically immersed in PBS at 100 rpm for up to 7 days at 37 °C. At each time point, the sample was removed and dried at 80 °C until no mass change was observed. The *R* value was calculated and the change in contact angle hysteresis (CAH) with DW and Fgn (2.6 mg/mL) was recorded. To examine their stability under shear stress, the samples were spun from 500 to 6000 rpm in PBS for 2 min, and the oil weight loss was monitored.

### Protein Adsorption Assay

The protein adsorption on SiR, AgR, SiO, and AgO surfaces was compared by immersing each sample (*n* = 3) in 2 mL of Fgn (2.6 mg/mL) at 37 °C at 100 rpm. After 1 day, the samples were taken out and gently rinsed with PBS, followed by ultrasonication (60 kHz) in 1 mL of 5 wt % sodium dodecyl sulfate (SDS) for 30 min. The protein concentration was determined using the bicinchoninic acid (BCA) method.^17^

### Bacterial Adhesion and Antibiofilm Assays

The antiadhesion efficacies of different samples were assessed against both *E. coli* and *P. mirabilis* in a 7-day model. In brief, bacteria were routinely cultured in tryptic soy broth (TSB) and grown to the log phase, centrifuged, and diluted to ∼10^7^ CFU/mL with PBS. Prior to the adhesion assay, all the samples were sterilized in 70% ethanol and placed in a 24-well plate. Each sample (*n* = 3) was incubated with 2 mL of the diluted bacterial culture at 37 °C and 100 rpm for up to 7 days. The bacterial suspension was refreshed every day and the number of attached viable bacteria was quantified by plate counting on day 1 and day 7. The adhered bacteria/biofilm was also stained with SYTO 9 and propidium iodide (2:1, v/v) and observed by fluorescence microscopy. To investigate the effect of host-secreted proteins (i.e., fibrinogen) on the antiadhesion activity, the samples were challenged with 2 mL of protein-bacteria suspensions (Fgn 2.6 mg/mL, bacteria ∼10^8^ CFU/mL in PBS) at 37 °C and 100 rpm. After 1 day of exposure, the samples were removed and gently washed with PBS to remove loosely adhered bacteria. The attached bacteria/biofilm were visualized by fluorescence microscopy and the surface coverage of the bacteria was analyzed using ImageJ.

### Bacterial Migration Assay

The ability of *P. mirabilis* to migrate over different types of catheter surfaces was investigated using a catheter bridge model described by Stickler.^18^ As shown in Figure 5a (presented later in this work), aliquots (10 μL) of the diluted log-phase bacterial cultures (∼10^5^ CFU/mL) were inoculated at the right edge of the channel and dried at 37 °C. After 30 min, 1 cm long segments of SiR, AgR, SiO, and AgO catheters (*n* = 3) were placed as bridges between the agar blocks to allow bacterial migration to the uninoculated halves of the plate. The plates were incubated at 37 °C for up to 48 h, and the time for *P. mirabilis* to across each type of catheter was recorded. To investigate the influence of Fgn binding on bacterial migration, all types of catheters were first immersed in Fgn solution (2.6 mg/mL) at 37 °C for 24 h and tested under the same conditions.

### Encrustation Assay

The antiencrustation properties of catheters were tested using a modified encrustation model, as reported by Jones and co-workers.^19^ In brief, 0.5-cm-long catheter segments (*n* = 3) were perpendicularly immersed in 3 mL of *P. mirabilis* (∼10^8^ CFU/mL) in artificial urine^20^ at 37 °C at 100 rpm for 24 h. The pH change at time points of 0, 2, 6, 12, and 24 h was monitored. The samples at each time point were taken out and the deposited crystals were observed using SEM.

### Cytotoxicity Assays

In vitro cytotoxicity against mouse fibroblast cells L929 (ECACC 85011425) was assessed using an MTT method by ISO 10993 standards. Fibroblast cells were routinely cultured to reach 80% confluency before seeding to a 96-well plate (100 μL/well, ∼10^5^ cells/well). Simultaneously, leachates from each material type (*n* = 6) were prepared by incubating the sterilized samples in supplemented EMEM (10% FBS, 100 mg/mL penicillin, and 100 mg/mL streptomycin) with a surface area:volume ratio of 3 cm^2^/mL at 37 °C for 24 h. After incubation, the media in each well was removed and replaced with 100 μL of corresponding extract-containing media and cultured for another 24 h. Subsequently, 50 μL of 3-(4,5-dimethylthiazol-2-yl)-2,5-diphenyltetrazolium bromide was added to each well and incubated for 4 h, followed by the addition of 500 μL of DMSO to dissolve the formazan. The absorbance was measured at 570 nm and relative cell viability was measured by comparison with the negative control (wells containing no sample extracts). The cells in each well were stained with phalloidin and DAPI according to the manufacturer’s instructions, and their morphologies were observed using a fluorescence microscope (Nikon 6D Live Cell Imaging Inverted Microscope, Nikon, Tokyo, Japan).

### Statistical Analysis

All data are presented as the mean ± standard deviation. A one-way ANOVA (Tukey’s post hoc) was performed to determine statistical significance, where values of *p* < 0.05 were considered significant and *p* < 0.01 were considered highly significant.

## Results and Discussion

### Surface Characterization and Silver Release

Silicone rubber due to its excellent mechanical property and biodurability has been widely used to manufacture urinary catheters. However, the bioinert and hydrophobic silicone catheters are prone to bacterial adhesion and the stiff surface may damage the urethra or the upper tract during insertion and replacement. To improve the antifouling and lubricating efficacies, in this study, commercial all-silicone urinary catheters were coated with AgNPs via a simple and cost-efficient wet chemistry method, followed by silicone oil infusion (Figure 1a).

Silver is one of the few FDA-approved antibacterial agents for catheter coatings and even low concentrations of Ag ions are enough to kill bacteria.^4^ The long-term efficacy of these silver-based coatings is directly dependent on the coating’s ability to provide sufficient, sustained Ag^+^ release. However, considering the cost, current commercial silver-based antibacterial catheters generally have a limited silver loading, and this results in short-term antibacterial efficacy. Moreover, the traditional silver coating often results in low silver utilization, since only the outermost layer of silver becomes oxidized and released into the surrounding environment. To address this limitation, a single layer of AgNPs was deposited on the SiR surfaces (Figures 1b and 1f). The size distribution of the AgNPs was relatively broad (∼3–76 nm) (Figure 1g), when compared with a previous report,^21^ and this was likely due to the higher reaction temperature that accelerated particle collision and caused random coalescence. The thickness of the AgNP layer was 42.2 ± 4.7 nm and the *R*_a_ value was 12.1 ± 3.3 nm. Over 7 days, the AgR catheters were capable of sustained release of Ag^+^ in PBS with a relatively constant daily release rate of ∼0.5 μg/cm^2^. The monodispersed AgNPs yield a greater contact surface in the liquid environment and will be more cost-efficient than traditional silver coatings.

**Figure 2:**
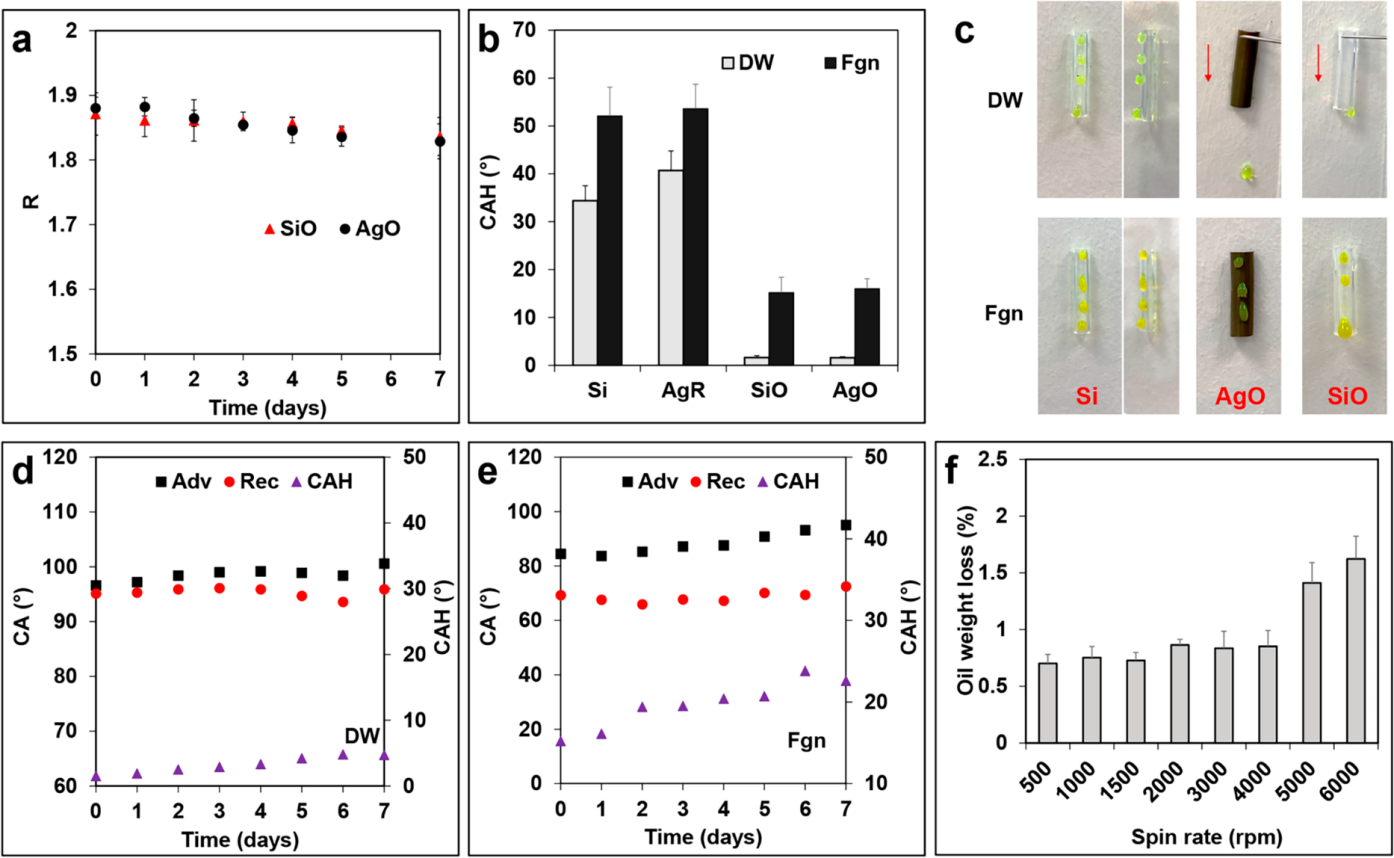
(a) Swelling ratios of SiO and AgO after immersing in PBS at 37 °C at 100 rpm for up to 7 days; (b) the CAHs of DW and Fgn on the different surfaces; (c) images showing DW and Fgn (with food color dye for observation) sliding on different surfaces at 90°; comparison of the CAHs of DW (d) and Fgn (e) measured on the AgO surfaces after immersing in PBS for up to 7 days; (f) oil weight loss ratio of AgO after spinning in PBS for 2 min at each spin rate from 500 to 6000 rpm (*n* = 6, bars represent standard deviation of the mean).

After silicone oil infusion, the nanoscale structure of the AgR surfaces was covered by silicone oil, yielding a flat and smooth liquid film on the catheter surface (Figure 1d). The thickness of the oil layer was 30.3 ± 6.1 μm. Notably, the AgO catheters exhibited a higher level of Ag^+^ release than the AgR catheters over the same test period (Figure 1h). Previous studies revealed that silicone oil could hinder the total hydration of the catheter surface, thus leading to a slower but sustained release of active molecules or drugs.^11,22^ In our case, the silver deposition resulted in a rougher surface which increased the surface hydrophobicity (water CA ≈ 116°, compared to Si (∼108°)) and retarded surface hydration as well as Ag^+^ release. For the AgO surface, the silicone oil effectively swelled the matrix network (Figure 2a), which may induce rapid leaching of loosely bonded AgNPs upon water flushing and simultaneously enable greater exposure to water. This was evidenced by the release data (Figure 1h) as the AgO showed an initial burst Ag^+^ release on the first day, followed by a sustained but slightly enhanced leaching of Ag^+^.

### Liquid Repellency and Surface Durability

The surface wettability of different surfaces was characterized by contact angle measurements. Compared with oil-infused surfaces, the bare SiR and AgR without a lubricant film showed typical hydrophobic properties, with a water CA of 108.2 ± 0.7° (SiR) and 116.2 ± 1.2° (AgR) respectively, and a high water CAH (SiR: 34.4° ± 2.6°; AgR: 40.7° ± 3.4°). Following infusion with silicone oil, the SiO and AgO surfaces showed a similar water CA of 95.5° ± 1.0° and extreme water repellency, as signified by very low CAH (∼1.6°) (Figure 2b), which confirms a lack of pinning and superslippery surfaces. However, upon contact with Fgn, the CAH increased on all the surfaces, despite the oil-infused surfaces still retaining greater repellency. This was further confirmed by the sliding experiment results (Figure 2c) as the bare catheters showed sticky Wenzel state wetting properties with water and Fgn droplets stuck on the surfaces (Movie 1 in the Supporting Information), whereas water droplets smoothly rolled off the oil-infused surfaces (Movie 2 in the Supporting Information). Unlike traditional surfaces, such as superhydrophobic surfaces, where CAH depends on liquid surface tension, such dependence is absent for SLIPS, because of the chemical homogeneity and physical smoothness of the liquid/liquid interface.^23^ Therefore, it is likely that the increased CAH and retention of Fgn on the oil-infused surfaces were attributable to chemical interactions between Fgn and silicone oil (Movie 3 in the Supporting Information; see further discussion in the later section, entitled “Fgn Adsorption and Bacterial Binding”).

For urinary catheters, the stability and longevity of surface-stabilized lubricant layers is a critical question for their antifouling properties. First, we investigated the loss of silicone oil from the SiO and AgO catheters in PBS under an estimated shear stress of 0.027 N/m^2^ for up to 7 days.^23^ The change in swelling ratios (*R*) with immersion time was monitored and no significant difference in *R* value was found between the SiO and AgO catheters throughout the test (*p* > 0.5). The *R* values of both groups were maintained relatively constant and only a slight decrease in *R* was noticed on day 7 (∼1.6%) (Figure 2a). The surface wettability change was monitored by CAH measurements with both DW and Fgn suspension. As shown in Figures 2d and 2e, the dynamic water and Fgn CAs on the surfaces of AgO remained nearly constant after flushing with PBS for 7 days. A slight increase in CAH was observed with both water (from 1.6° to 4.7°) and Fgn (from 15.8° to 22.6°) on day 7, which is likely to be associated with the oil loss that causes a rougher surface. Nevertheless, the low CAH after 7 days of immersion indicated that the AgO catheters can maintain significant stability in a flowing water environment. Early studies on SLIPs revealed that the fluidic nature of the lubricating layer enables the silicone oil to flow toward the damaged area by surface-energy-driven capillary action, and spontaneously refills the physical voids.^24^ To verify such self-healing properties, the AgO catheters were centrifuged in PBS from 500 to 6000 rpm for 2 min and the oil loss was calculated. As shown in Figure 2f, the oil loss was negligible (<1%) following centrifugation below 4000 rpm, and slightly increased to ∼1.5% with the spin rate increased to 5000 rpm.

### Bacterial Adhesion

**Figure 3:**
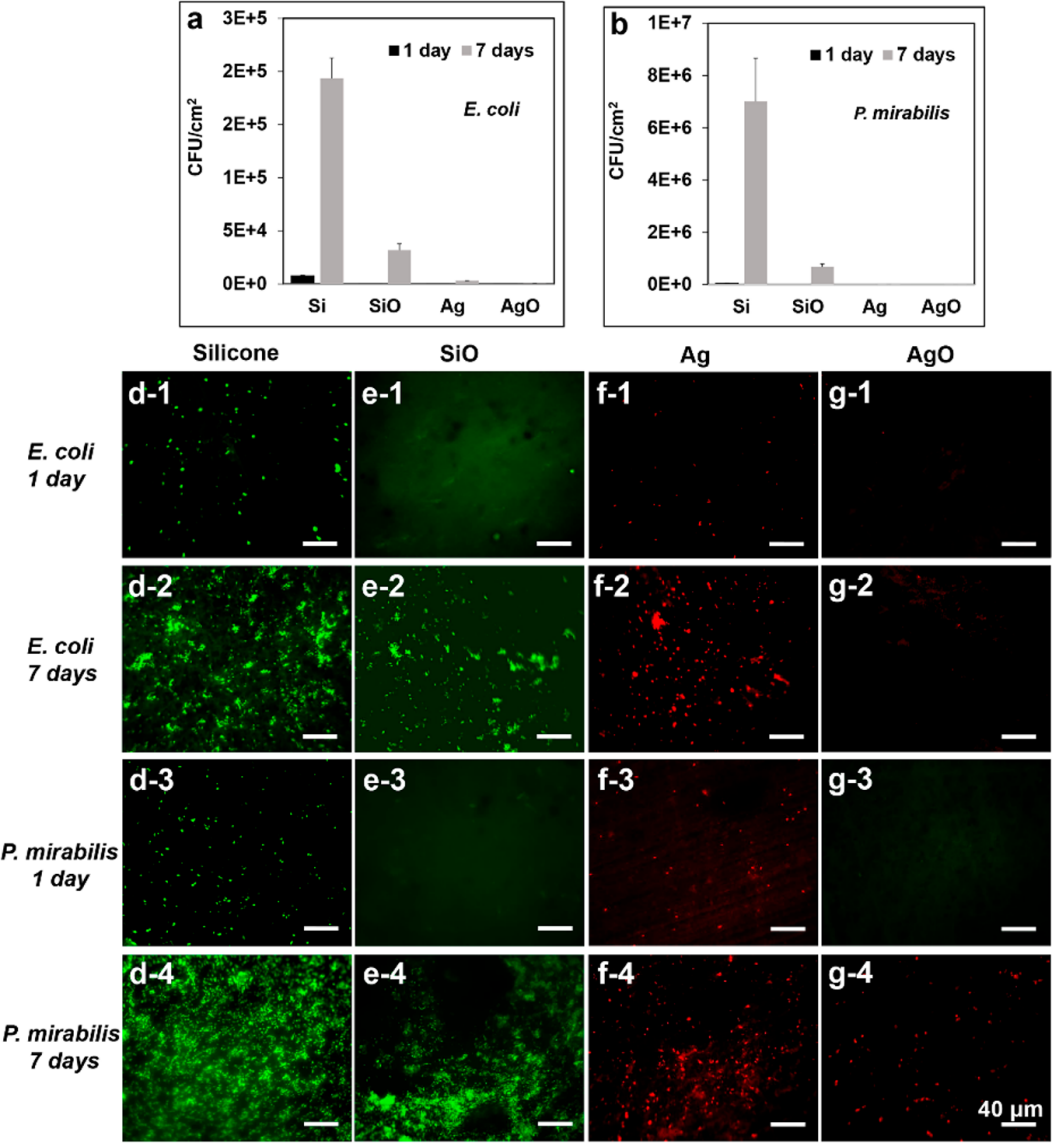
Quantitative counts of viable (a) *E. coli* and (b) *P. mirabilis* cells adhering to different surfaces after 1 day and 7 days of incubation; live/dead assay: (d–g) fluorescence microscope images of *E. coli* and *P. mirabilis* on different surfaces after 1 day and 7 days coculture. Typical images are shown from one of several examinations (*n* = 6, bars represent the standard deviation of the mean, scale bars correspond to 40 μm).

**Figure 4:**
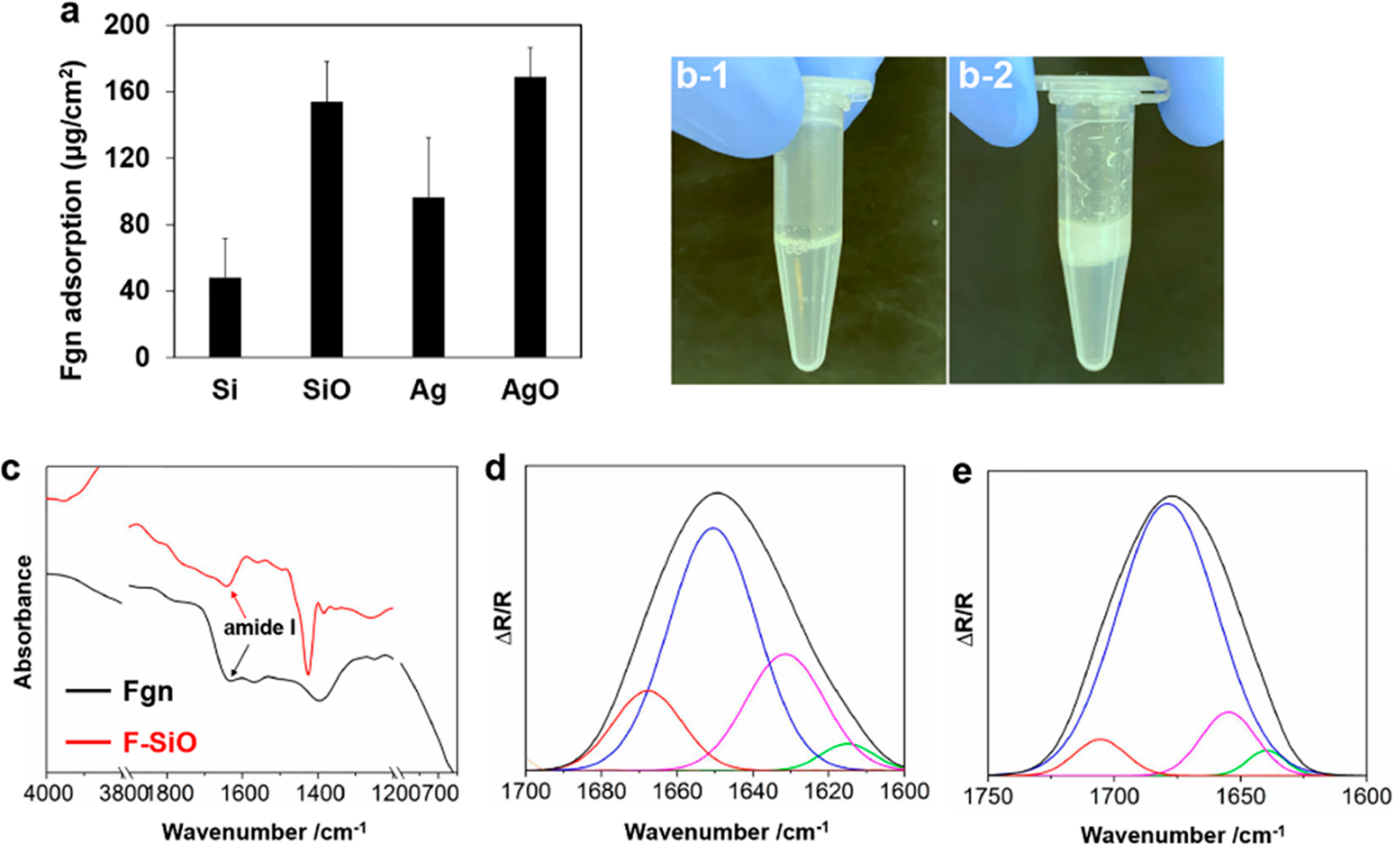
(a) Fgn adsorption on different surfaces after 24 h; images of Fgn suspension (b-1) before and (b-2) after contact with silicone oil; (c) FTIR spectra of Fgn before and after contact with silicone oil; (d) deconvolution of the amide I absorption band of fibrinogen with Gaussian line function, before (d) and after (e) contacting silicone oil, secondary structures of the component bands were assigned as follows: (green) β-sheet, (pink) α-helix, (blue) and (red) β-turns (*n* = 6, bars represent standard deviation of the mean).

The efficacy of SiO and AgO catheters to resist bacterial adhesion was assessed with two common uropathogens: *E. coli* and *P. mirabilis*. After 24 h of immersion, the SiO catheter demonstrated excellent antiadhesion activity against both species, when compared with the control SiR catheters, with almost no viable cells attached (Figures 3a and 3b). This could be ascribed to its ultralow CAH that inhibited bacterial cells to establish stable and strong interactions with the liquid-like surfaces, resulting in detachment from the surface by the action of very low wall shear stress (which corresponds to similar wall shear stress in catheters).^10^ However, significant bacterial attachment was observed on the SiO surfaces (Figures 3e-2 and 3e-4) after 7 days, despite the SiO still reducing over 80% of *E. coli* and 90% of *P. mirabilis* adhesion. The bacterial cells were able to penetrate the lubricant layer and proliferate,^24^ as evidenced by observing no drastic decrease in CAH (water CAH = 6.4°). These results indicated that the catheters with silicone-oil infusion alone were only able to provide short-term protection against bacterial adhesion. In comparison, the AgR and AgO catheters demonstrated superior antibacterial activities throughout the test period, reducing ∼99% of viable bacterial adhesion after 7 days. For the AgR surfaces, nearly all the attached cells were dead, but the number of attached bacteria significantly increased over time. Considering the hydrophobic character of the AgR catheters (Figure 1h) and daily refreshment of bacterial suspension, the relatively slow release of Ag^+^ from the AgR surfaces may not kill all the bacteria in the surrounding environment and lead to increased bacterial attachment with time. Upon close contact with AgNPs, the proliferation of attached bacteria was effectively inhibited as no cell clusters or biofilms were observed, even after 7 days. However, accumulated dead cells may eventually cover the AgR surfaces and shield the bacteria from the underlying AgNPs, leading to biofilm formation over a longer period. In contrast, the AgO surfaces exhibited the lowest bacterial adhesion, further reducing 85.7% of *E. coli* and 64.4% of *P. mirabilis* adhesion, compared to the AgR surface on day 7. This is likely due to enhanced leaching of Ag^+^ (Figure 1h) that facilitates rapid bacterial inactivation. Taken together, enhanced Ag^+^ release from the AgO catheters is an essential contributor to the long-term suppression of bacterial colonisation.

### Fgn Adsorption and Bacterial Binding

Recent findings revealed that urinary catheterization induces fibrinogen release into the bladder as part of the inflammatory response, and the proteins could deposit on the catheter surfaces facilitating the binding of certain types of uropathogens, enhancing bacterial colonization and biofilm formation during CAUTIs.^25,26^ Flores-Mireles et al.^27^ reported that *Enterococci* bacteria (e.g., *Enterococcus faecalis*) could bind fibrinogen via the endocarditis- and biofilm-associated (Ebp) pilus and use it as a food source for producing proteases. Therefore, in addition to resisting bacterial adhesion, a surface that is capable of inhibiting protein adsorption would be beneficial for reducing instances of CAUTIs. In this work, Fgn adhesion on different surfaces was examined at pH 7.4 for 24 h. Surprisingly, the oil-infused coatings showed significantly higher levels of Fgn binding (*p* < 0.05) than bare SiR or AgR surfaces (Figure 4a). The SiO and AgO surfaces had similar protein absorption of ∼155 μg/cm^2^, which was ∼3 times higher than the SiR surfaces (∼48 μg/cm^2^). As evident from the dynamic contact angle analysis (Figures 2c and 2e), the oil-infused surfaces also demonstrated significant protein retention. Considering their supersmooth surfaces (Figure 1d), the enhanced Fgn adsorption and retention were likely due to the intermolecular interaction between the silicone oil and Fgn that induced rapid protein aggregation and binding to the surface.^28^

To verify this hypothesis, Fgn (2.6 mg/L in PBS) was mixed with silicone oil at room temperature and vortexed for 30 s, followed by standing for 5 min at 37 °C. As seen in Figure 4b-2, the Fgn rapidly aggregated into cloud-like condensates after contact with silicone oil and attached to the tube walls, exhibiting enhanced viscosity as compared with the control (Figure 4b-1). To investigate the conformational change of the Fgn, whole infrared spectra were compared, and second derivative spectral analysis was conducted to locate the position of the overlapped components of amide I and to assign them to different functional groups. Curve fitting was performed by setting the number of component bands found by second-derivative analysis with a fixed bandwidth (12 cm^–1^) and Gaussian profile.^29^ As seen in Figure 4c, the peak position of the amide I band red-shifted from 1630 cm^–1^ to 1642 cm^–1^ in the presence of silicone oil, indicating a conformational change that resulted in aromatic side chains moving to a less polar environment, similar to the aggregation process.^30^Figures 4d and 4e show that β-sheet, α-helix, and β-turns were the three dominating secondary structures in Fgn. Upon coming into contact with silicone oil, both the β-sheet (from 3.72% to 2.88%) and α-helix (from 24.4% to 10.5%) content decreased while the β-turns (from 71.8% to 86.6%) content significantly increased. In agreement with previous studies,^30,31^ a possible assumption is the conversion of β-sheet and α-helices into β-turns, which suggests the partial denature of proteins with a loss of helical secondary structures. This may explain the rapid Fgn adhesion on the oil-infused surfaces as silicone oil facilitated protein structure rearrangements and accelerated the binding of proteins onto the surface.

**Figure 5:**
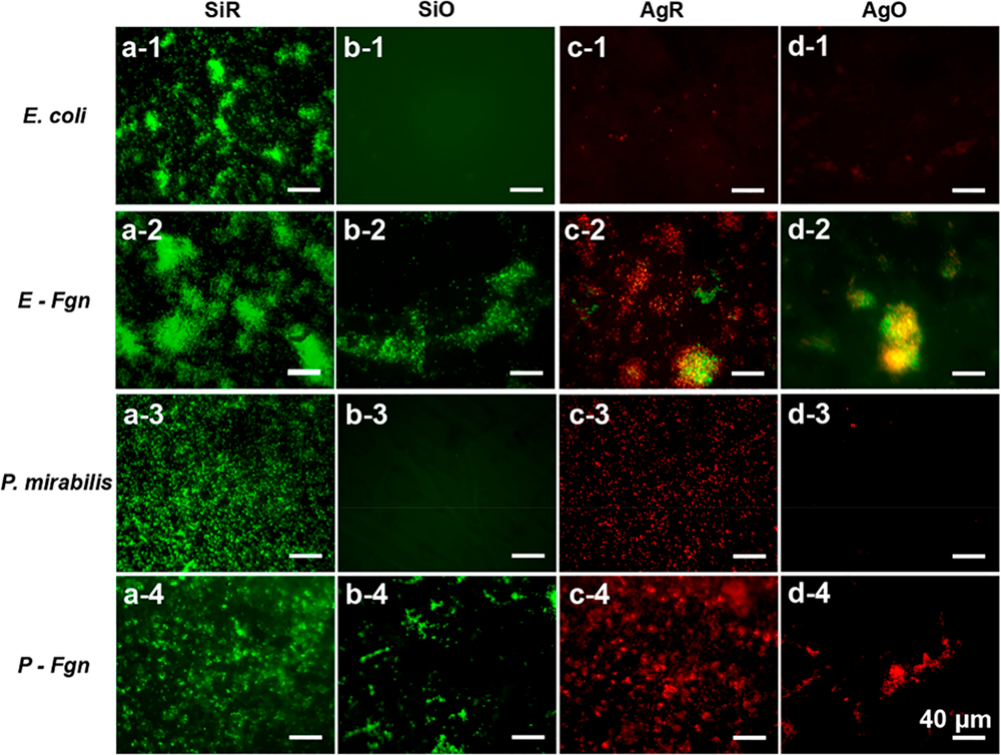
Fluorescence microscope images of *E. coli* and *P. mirabilis* on different surfaces after 1-day coculture. Typical images are shown from one of several examinations (scale bars correspond to 40 μm).

To investigate the effect of Fgn adsorption on bacterial adhesion, the samples were challenged with a protein-bacteria model for 24 h, and the adhered cells were observed by fluorescence microscopy. As shown in Figure 5, the presence of Fgn in bacterial suspensions induced a significant change in bacterial adhesion on all types of surfaces. The oil-infused surfaces exhibited the lowest bacterial adhesion in the absence of Fgn for both strains, and the results are consistent with Figure 3. After introducing Fgn into the medium, a significant increase in bacterial colonization was observed on both SiO and AgO surfaces. Note that the attached bacteria were not uniformly distributed on the oil-infused surfaces where cells were mostly binding within a framework (Figures 5b-2, 5d-2, 5b-4, and 5d-4). According to Andersen et al.,^15^ Fgn deposition on catheters is not uniform and bacteria bind more extensively to catheters with Fgn present. Similar phenomena were also observed with the bare SiR and AgR surfaces. In comparison, the attached bacteria without Fgn codeposition were nearly isolated and monodispersed. For the silver-releasing samples, their surfaces retained potent antibacterial activities in the presence of Fgn as most of the attached bacteria were killed. However, as evident from the surviving bacteria on the AgR and AgO surfaces, the gradual accumulation of proteins and biomass may eventually shield the bacteria from the underlying silver, creating a microenvironment for catheter colonization.

To investigate the effect of oil thickness on bacterial adhesion, we further prepared AgO samples with a coating thickness of ∼14 μm (AgO-1). The results (Figure S1a in the Supporting Information) showed that a thinner oil layer resulted in a slower Ag^+^ release in PBS but similar Fgn adsorption (Figure S1b in the Supporting Information). In the presence of Fgn, the Ag^+^ release from all the samples was retarded, indicating that absorbed protein hindered silver release, and this may further block the interaction of antibacterial Ag^+^ with bacteria. To verify this assumption, the effect of oil thickness on biofilm adhesion was assessed by comparing the number of viable cells attached to different surfaces. As seen in Figure S1c in the Supporting Information, all the surfaces (AgR, AgO, and AgO-1) showed a similar bacterial adhesion (*p* > 0.05) and this result is consistent with the ICP results in Figure S1a. These results indicated that the silicone oil layer facilitated Fgn adsorption on the AgO and AgO-1 surfaces, regardless of oil thickness, and the deposited Fgn blocked the interaction of silver with the bacteria, resulting in a compromised antibiofilm activity.

### Bacterial Migration and Encrustation

**Figure 6:**
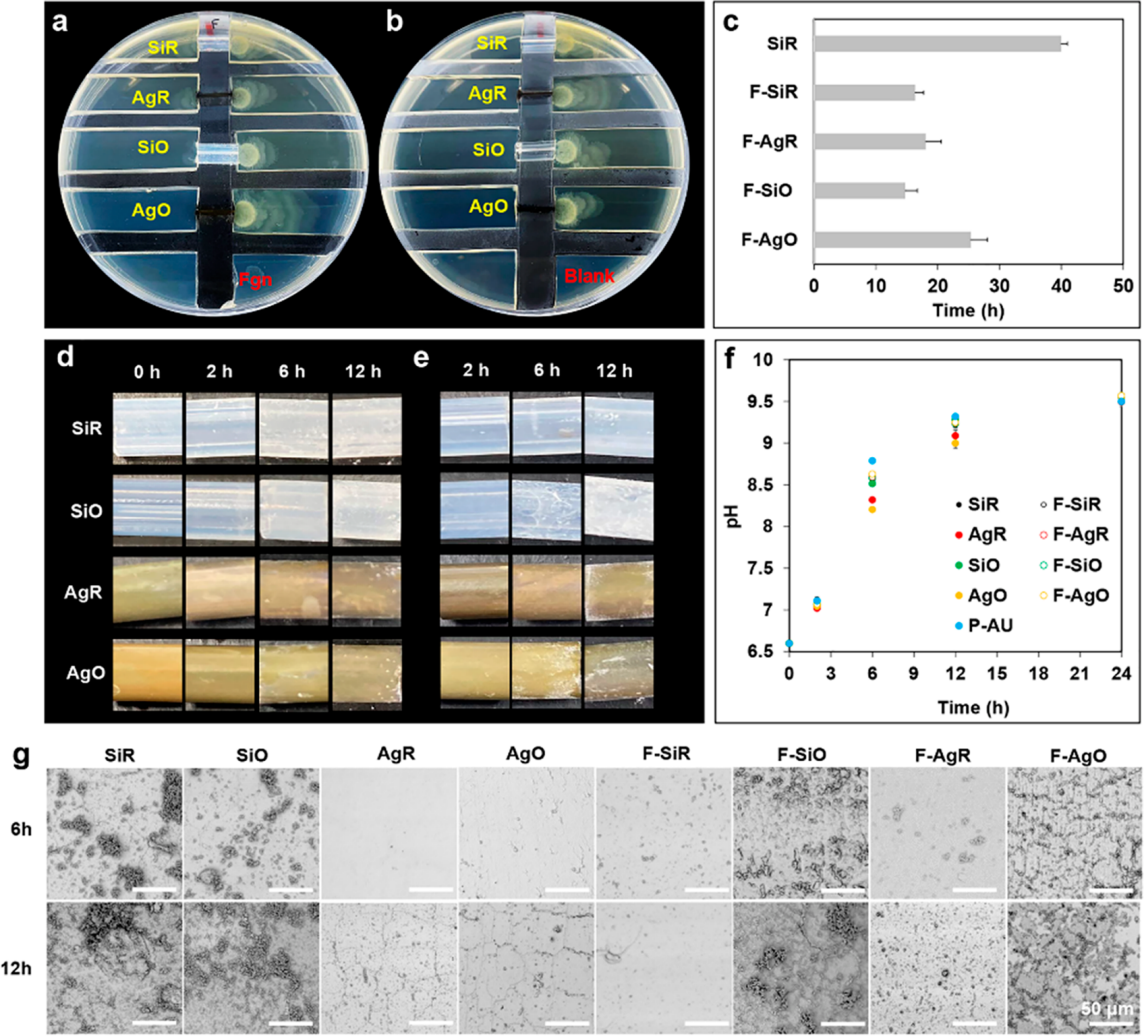
*P. mirabilis* migrating over 1 cm sections of (a) bare catheters and (b) Fgn-pretreated catheters after 24 h; (c) migration time for each catheter (within 48 h); encrustation formation over time on (d) bare catheters and (e) Fgn-pretreated catheters; (f) pH change with time; (g) SEM images of different catheter surfaces after encrustation test for 6 and 12 h (*n* = 3, bars represent standard deviation of the mean; scale bars correspond to 50 μm).

Clinical studies indicate that bacteria can initiate infections by migrating along either the external surface or the lumen of the catheter into the bladder.^32^*P. mirabilis*, a Gram-negative rod-like bacterium, is the main pathogen causing complicated CAUTIs, because of its swarming motility and urease-producing activity. *P. mirabilis* can transform from small swimming bacilli into highly flagellated swarmer cells after attaching the catheter surface and swarm rapidly over the catheter into the bladder and kidney, and this is accompanied by a substantial increase in the production of urease, resulting in the rise in urinary pH and formation of crystalline biofilms.^18,33^ Considering the weak and intermittent urine flow over the catheter surfaces in clinical settings, the ability of different catheters to resist bacterial migration was assessed using a bridge model and the effect of Fgn binding on migration rate was also investigated. As seen in Figures 6a and 6b, the Fgn binding dramatically accelerated bacterial migration over all types of catheters. *P. mirabilis* swarmed over 1 cm sections of the F-SiO catheters in ∼14.6 ± 2.1 h, followed by F–Si (16.3 ± 1.4 h), F–Ag (18.1 ± 2.3 h), and F-AgO (25.2 ± 2.6 h) catheters. In comparison, *P. mirabilis* migrated over the bare SiR catheters within ∼40 h but failed to migrate across AgR, SiO, and AgO catheters within 48 h (Figure 6c). These observations suggest that surface binding with Fgn may facilitate the spread of crystalline biofilm over catheter surfaces.

To verify this hypothesis, the antiencrustation performance of the catheters was assessed using an in vitro model. Crystal deposition was not observed on all types of catheters within 2 h of incubation (Figures 6d and 6e) and the artificial urine remained clear with a neutral pH value of 7.1 (Figure 6f). For the bare catheters, a significant rise in urinary pH was noticed after 6 h and crystalline deposits were formed on both SiR and SiO surfaces (Figure 6g). The AgR and AgO surfaces were free from crystal deposition and the corresponding urine pH values were slightly lower, when compared with that of SiR and SiO. The released Ag^+^ effectively retarded bacterial adhesion, but note that not all the bacteria were killed at this stage as the urine pH continued to rise with time. Because of the lack of antibacterial activity, the SiR and SiO surfaces were covered with a layer of dense crystalline biofilms after 12 h while only trace amounts of crystals were formed on the AgR and AgO surfaces. The AgO surfaces exhibited the lowest crystalline biofilm coverage, which is consistent with the antiadhesion results in Figure 3. However, for the oil-infused catheters, the Fgn binding significantly altered their antiencrustation properties as crystals deposited more rapidly on the SiO and AgO catheters by 6 h when compared with the bare catheters (Figures 6d and 6e). As seen in Figure 4b, the oil-infused surfaces attracted significant higher levels of Fgn adsorption, compared with bare catheters, and the results further revealed that surface binding with Fgn could facilitate biofilm formation and the silicone-oil-infused catheters were unable to resist Fgn-associated encrustation in vitro. Moreover, the Ag^+^ release from the F-AgR and F-AgO catheters was likely to be hindered due to the adsorbed Fgn as no significant difference in urine pH was observed between the Fgn binding groups (Figure 6f).

### Cytotoxicity Assay

**Figure 7:**
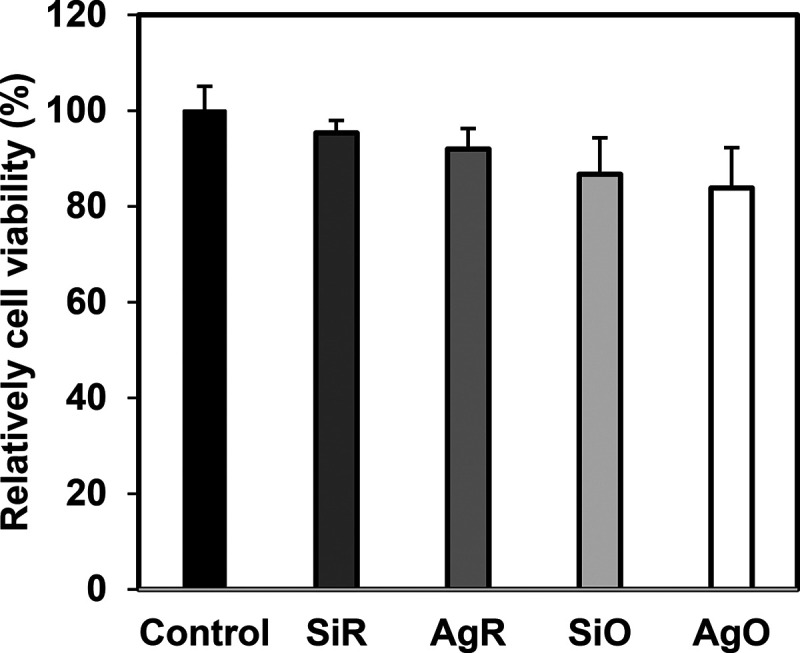
Relative cell viability after 24-h exposure to various sample extracts (*n* = 6, bars are standard error of the mean).

**Figure 8:**
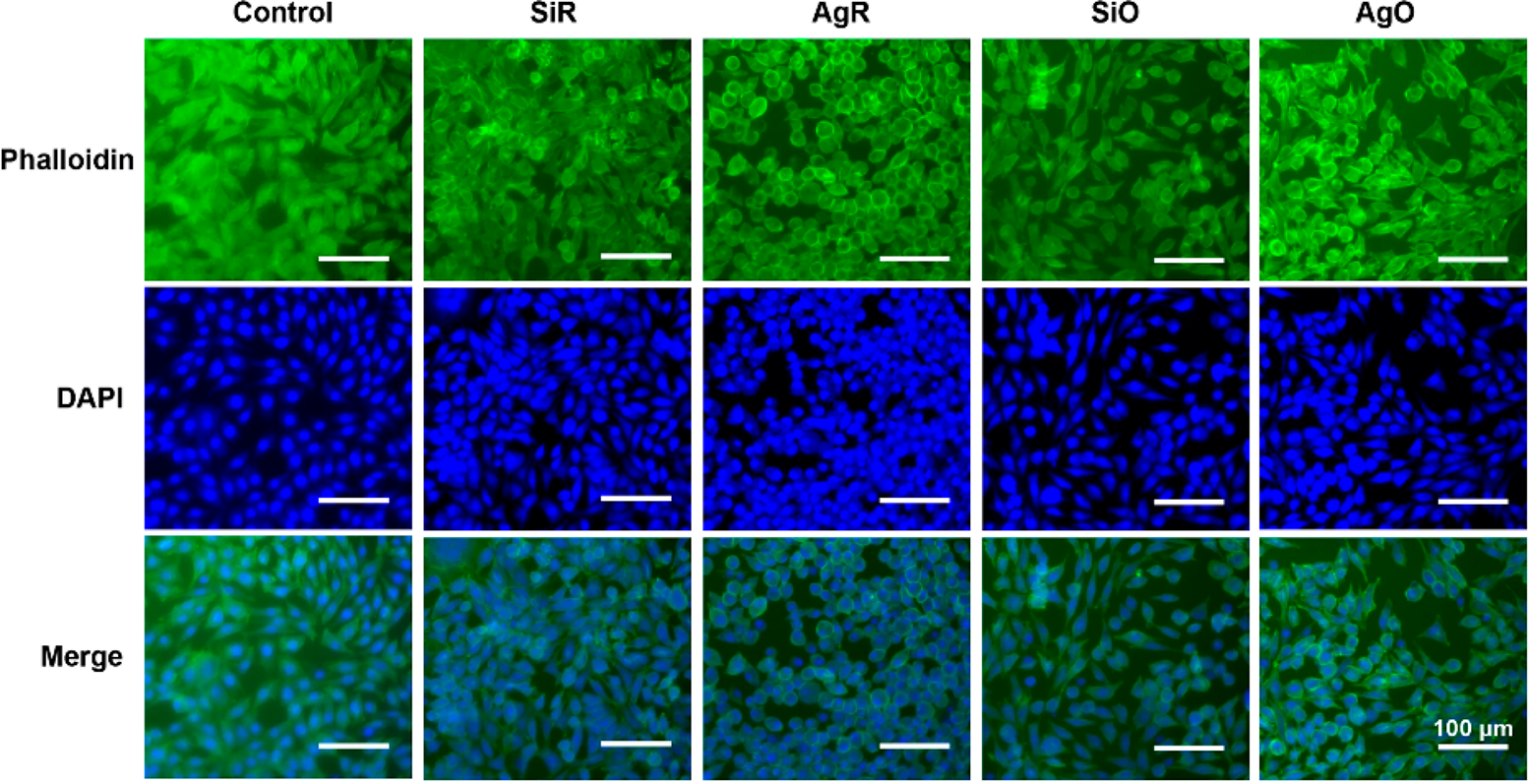
Fluorescence images of L929 cells after 24 h of incubation with different sample exacts (scale bar corresponds to 100 μm). Typical images are shown from one of several examinations.

Silver has long been recognized as a double-edged sword, because it can exert a broad-spectrum bactericidal activity at very low concentrations but become cytotoxic toward human cell lines at high levels.^34^ Moreover, despite being considered nontoxic, silicone oil has been reported to be toxic to cultivated corneal endothelium cells.^35^ In this study, the toxicity of the coated catheters was evaluated with L929 mouse fibroblast cells in vitro. As shown in Figure 7, the SiR catheters after silicone oil infusion showed a decrease in viable cells (*p* < 0.05). A similar finding has also been reported by Handa et al.^25^ A recent study revealed that most silicone oil contains chemical impurities (e.g., low-molecular-weight components) that may diffuse into cells and cause cytotoxicity,^36^ but definitive clinical evidence of silicone oil toxicity is still lacking. The silver deposition on either SiR or SiO catheters induced no significant decrease in viable cells (*p* > 0.05) despite the fact that the AgO catheters released a relatively higher level of Ag^+^ within 24 h (Figure 1h). Considering the low silver loading on catheters (Figure 1c), the overall toxicity elicited toward mouse fibroblast cells is favorably negligible. Figure 8 shows the morphologies of L929 cells cultured with different sample extracts after 24 h. The cells in control, SiR, and SiO groups exhibited healthy spindle-like morphology and the cell nuclei indicated by DAPI staining showed full integrity. Several ovoid-shaped cells were observed in the AgR and AgO groups, but no obvious morphological damage was noticed. These findings suggest that all the samples did not exert significant toxicity at this stage. However, further evaluation may be required to understand long-term cellular responses toward the silver-containing surfaces.

## Conclusions

A deeper understanding of the pathogenesis of CAUTIs is critical for developing a new and effective anti-infection urinary catheter to address the current challenges. In this work, we describe a simple procedure to fabricate a liquid-like slippery silicone-oil-infused silver-releasing coating for urinary catheters. The as-prepared catheters demonstrated superior stability and durability in aqueous conditions, due to inherent self-healing properties and exhibited outstanding long-term antibacterial and antiadhesion properties against *E. coli* and *P. mirabilis*, because of outstanding water repellency and enhanced silver release. In the presence of clinical-relevant Fgn, the silicone-oil-infused surface could trigger protein conformation and accelerate protein binding, which, in turn, resulted in a loss of liquid repellence and enhanced bacterial colonisation. Our in vitro results showed that the Fgn binding could facilitate *P. mirabilis* migration along the catheter surfaces and cause accelerated encrustation deposition and spread of a crystalline biofilm over the catheter surfaces. Despite the coated catheters showing good biocompatibility, the silicone-oil-infused catheters may not hold significant potential for the development of next-generation anti-infection urinary catheters, unless catheter-associated bladder inflammation could be effectively avoided.

## Supporting Information Available

Measurement oil thickness, the effects of oil thickness on silver ion release, Fgn adsorption, and bacterial adhesion (PDF)
Movie 1 (MP4)
Movie 2 (MP4)
Movie 3 (MP4)
