# Supplementary material for: Fibrinogen Deposition on Silicone Oil-Infused Silver-Releasing Urinary Catheters Compromises Antibiofilm and Anti-Encrustation Properties
Source: Langmuir. 2023 Jan 20;39(4):1562–72. doi: 10.1021/acs.langmuir.2c03020 (PMC9893812; doi:10.1021/acs.langmuir.2c03020)
Supplement: Supplementary file 1 — la2c03020_si_001.pdf [file la2c03020_si_001.pdf]

Fibrinogen Deposition on Silicone Oil-Infused Silver-Releasing Urinary Catheters Compromises Antibiofilm and Anti-Encrustation Properties

Shuai Zhang [1, *], Xiao Teng [1], Xinjin Liang [2, 3], Geoffrey Michael Gadd [3, 4], Colin Peter McCoy [1], Yuhang Dong [5], Yimeng Wang [5], Qi Zhao [5]

[1] School of Pharmacy, Queen's University Belfast, BT9 7BL, Belfast, UK;

[2] School of Life Sciences, University of Dundee, DD1 5EH, Dundee, UK;

[3] School of Mechanical and Aerospace Engineering, Queen's University Belfast, BT9 AG, Belfast, UK;

[4] State Key Laboratory of Heavy Oil Processing, Beijing Key Laboratory of Oil and Gas Pollution Control, China University of Petroleum, Beijing 102249, China;

[5] School of Science and Engineering, University of Dundee, DD1 4HN, Dundee, UK.

[*] Corresponding author. E-mail: shuai.zhang@qub.ac.uk

1. As the oil diffuses into the silicone catheter, measuring the weight of the swelling oil cannot be used to find the thickness of the surface oil layer. By assuming that the silicone catheters swell isotropically, however, measurements of weight and volume before swelling, after swelling, and after wiping could be used to approximate the layer thickness and volume of infused oil. By solving a cubic polynomial function, the oil thickness can be calculated.

In brief, the weight (m_1) of the original catheter was measured. The weight (m_2) and the dimensions (x,y,z) of the swollen catheter after wiping were also measured. A total of 6 replicates were used for the measurements. Due to the nature of physical absorption, the oil thickness (t) is assumed uniform in all directions. The oil thickness is then determined using the following equation:

$$\frac{m1 - m2}{\rho} = (x+2t)(y+2t)(z+2t) - xyz \qquad \text{(a)}$$

where x, y, z are the length, width, and height of the swollen catheter, respectively. The oil density (ρ) used in this study was 0.93g/cm^3. In this study, the measured oil thickness was estimated to be 30.3 ± 6.1 µm.

2. To investigate the effect of oil thickness on bacterial adhesion, we further prepared AgO samples with a coating thickness of ~ 14 µm (AgO-1). The coating thickness was calculated using equation a. The release of Ag^+ in neat PBS and Fgn-supplemented PBS was determined by ICP- OES, Fgn adhesion was assessed by the BCA method, and effect of Fgn adsorption on antibiofilm activity was determined by a plate count method after 24 h and compared with AgR and AgO (thickness of ~ 30 µm).

As seen in Figure S1a, the thinner oil layer in the AgO-1 resulted in a slower release of Ag^+ in PBS when compared with the AgO. According to equation a, the reduced oil adsorption in AgO-1 led to a lower swelling ratio in all directions when compared with the AgO, and this may hinder the leaching of loosely bonded AgNPs upon water flushing. In the presence of Fgn, the release of Ag^+ from all the samples was retarded, indicating that absorbed protein hindered silver release and this may further block the interaction of antibacterial Ag^+ with bacteria. Further, we assessed the effect of oil thickness on Fgn adhesion. As shown in Figure S1b, no significant difference ($p>0.05$) in Fgn adhesion was observed between the AgO and AgO-1 samples. This indicated that the silicone oil layer was stable during the

test and could facilitate Fgn adsorption regardless of oil thickness. To investigate the effect of oil thickness on biofilm adhesion, the number of viable cells attached to different surfaces was counted and compared. For both strains, no significant difference ($p>0.05$) was noted in the number of attached viable cells on the AgR, AgO and AgO-1 surfaces. This result is consistent with the ICP results in Figure S1a. Overall, the silicone oil layer facilitated Fgn adsorption on the AgO and AgO-1 surfaces regardless of oil thickness and the deposited Fgn blocked the interaction of silver with the bacteria, resulting in a compromised antibiofilm activity.

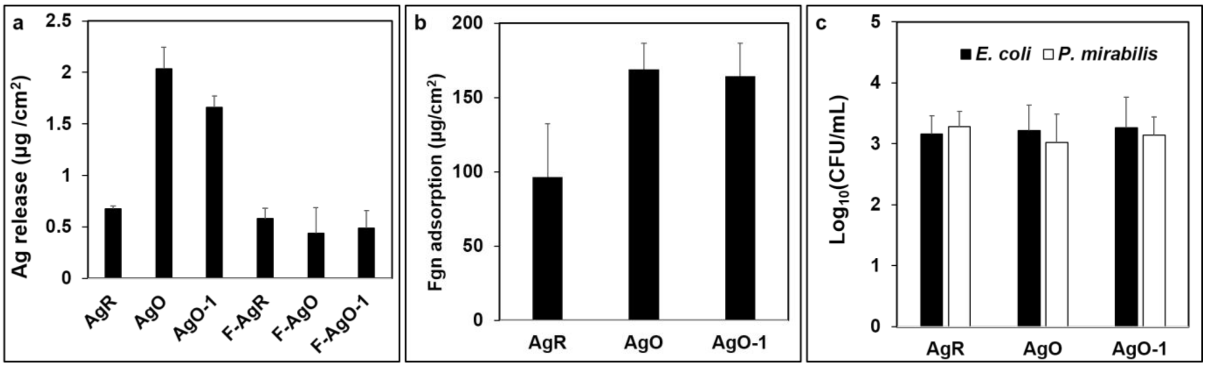

Figure S1. (a) silver release profiles from AgR, AgO, and AgO-1 in PBS and Fgn suspension over time; (b) Fgn adsorption on Ag, AgO, and AgO-1 surfaces after 24 h; (c) Quantitative counts of viable *E. coli* and *P. mirabilis* cells adhering to Ag, AgO, and AgO-1 surfaces after 24 h of incubation ($n = 6$, bars represent standard deviation of the mean).
